# Unbiased Identification of Patients with Disorders of Sex Development

**DOI:** 10.1371/journal.pone.0108702

**Published:** 2014-09-30

**Authors:** David A. Hanauer, Melissa Gardner, David E. Sandberg

**Affiliations:** 1 University of Michigan, Department of Pediatrics & Communicable Diseases, Ann Arbor, Michigan, United States of America; 2 University of Michigan, Pediatrics & Communicable Diseases and Child Health Evaluation & Research (CHEAR) Unit, Ann Arbor, Michigan, United States of America; National Taiwan University, Taiwan

## Abstract

Disorders of sex development (DSD) represent a collection of rare diseases that generate substantial controversy regarding best practices for diagnosis and treatment. A significant barrier preventing a better understanding of how patients with these conditions should be evaluated and treated, especially from a psychological standpoint, is the lack of systematic and standardized approaches to identify cases for study inclusion. Common approaches include “hand-picked” subjects already known to the practice, which could introduce bias. We implemented an informatics-based approach to identify patients with DSD from electronic health records (EHRs) at three large, academic children’s hospitals. The informatics approach involved comprehensively searching EHRs at each hospital using a combination of structured billing codes as an initial filtering strategy followed by keywords applied to the free text clinical documentation. The informatics approach was implemented to replicate the functionality of an EHR search engine (EMERSE) available at one of the hospitals. At the two hospitals that did not have EMERSE, we compared case ascertainment using the informatics method to traditional approaches employed for identifying subjects. Potential cases identified using all approaches were manually reviewed by experts in DSD to verify eligibility criteria. At the two institutions where both the informatics and traditional approaches were applied, the informatics approach identified substantially higher numbers of potential study subjects. The traditional approaches yielded 14 and 28 patients with DSD, respectively; the informatics approach yielded 226 and 77 patients, respectively. The informatics approach missed only a few cases that the traditional approaches identified, largely because those cases were known to the study team, but patient data were not in the particular children’s hospital EHR. The use of informatics approaches to search electronic documentation can result in substantially larger numbers of subjects identified for studies of rare diseases such as DSD, and these approaches can be applied across hospitals.

## Background

Disorders of sex development (DSD) represent a prototype for rare diseases research. Patients with DSD have congenital conditions in which development of chromosomal, gonadal, or anatomic sex is atypical [1]. The various conditions subsumed under the umbrella term DSD are individually rare but, in the aggregate, have an estimated incidence of 0.1 to 0.5% of live births [2]. Substantial controversy exists regarding “best practices” in DSD; debate surrounds principles guiding gender assignment decisions, genital or gonadal surgery and their timing, hormone replacement protocols, and strategies toward educating patients and others about details of the medical condition [3]–[5].

A major barrier to an improved understanding of the relationships between clinical practice and outcomes include small, incomplete, or selected (i.e., “hand-picked”) study samples. Indeed, the ability to generalize findings from studies of DSD patients has been limited by the lack of systematic and standardized approaches to identify patients for inclusion, and selection bias has been described as one of six “General problems of outcome studies in Disorders of Sex Development” [6]. Typical cohort ascertainment approaches include (1) clinician nomination (e.g., physician or other health care provider) or use of informal registries (e.g., patient lists) maintained by individual clinicians [7]–[9], (2) invitations to members of DSD peer support organizations [9], [10], and (3) reviewing clinic schedules during the recruitment phase for recognizable patient names or relevant diagnoses [11]. Many published studies do not consider how these approaches might impact interpretation of the results [12], [13].

Perhaps nowhere are these shortcomings more relevant than in the study of psychosocial and psychosexual outcomes in DSD-affected persons, where many of the factors that might lead a research team to select a patient for study inclusion (e.g., predicted willingness to participate, relative ease to locate, etc.) are likely correlated with psychological characteristics and thereby result in a potentially biased sample [14]. More robust and unbiased approaches are, therefore, necessary to reduce the lack of representativeness that often results from less rigorous cohort ascertainment protocols.

Electronic health records (EHRs) have the potential for supporting improved methods of identifying eligible study subjects for rare diseases, but significant challenges remain. For many rare diseases, easily extractable structured data elements (e.g., ICD-9 codes) do not provide the discrimination necessary for accurate cohort identification. For example, there is no specific ICD-9 code for DSD, and inferences must often be made based on a variety of candidate codes and other clinical attributes found in the free text narrative documents. Additionally, assignment of ICD-9 codes for a variety of disorders has been shown to be inaccurate [15]–[19]. This is not surprising because for many disorders, including those classified as DSD, the diagnoses are often made over time, yet the initial ICD-9 codes were assigned when diagnostic uncertainty was high. For example, it is not uncommon to find a patient with a coded diagnosis based on the initial presentation of “hypospadias,” whereas the final and more accurate diagnosis of “partial androgen insensitivity syndrome” is only mentioned in the narrative clinical notes that are created subsequent to the initial encounter.

Substantial amounts of clinical data can only be found in the free text narrative portions of clinical documents [20], [21]. Such documents are often created by clinicians to record the salient details of a clinical encounter and contain many details that are difficult to express using more structured data entry methods. In the case of DSD, these details can include visual descriptions of the genitalia that are easiest to express in an unstructured, free text, narrative format. They are often created via typing directly into an EHR system or through dictation and subsequent transcription [22]. These narrative documents remain the primary means of communication between clinical providers [23]. Because of their central role in communication and capture of important clinical details, these free text clinical documents must often be read to identify features necessary to make an accurate assessment about study eligibility. Accordingly, simply having the data available in electronic format does not necessarily lead to an improved capacity for cohort identification because many EHRs provide inadequate tools for improving efficiency by searching. This has resulted in an incongruity wherein the data may be available to yield a more complete sample for research, but the ability for research teams to make use of the data remains limited [24].

Computational approaches for information extraction and cohort identification can involve the use of natural language processing (NLP) [25], but the broader application of NLP is impeded by the technical expertise required for implementing the systems and the complexity of applying the algorithms across multiple institutions [26]–[28]. Other informatics approaches that do not involve traditional NLP may be easier to implement and can still help research teams access the data “locked” in the medical record [29]–[31] as well as provide reliable methods for cohort discovery in electronic clinical documentation across diverse clinical environments.

Here we describe one such approach for rare disease cohort discovery that was tested at three large academic medical centers for the purpose of identifying complete cohorts of DSD-affected patients. We show that the method yields substantially larger numbers of patients compared to traditional cohort identification approaches, and that the approach is applicable across multiple institutions using different EHRs. By doing so, we demonstrate that similar approaches are potentially within reach of any medical institution with an EHR interested in identifying representative cohorts for rare diseases research.

## Methods

### Study Context

As part of a broader health-related quality of life study, we sought to identify a complete cohort of patients with DSD from three large, tertiary, academic children’s hospitals, referred to here as Hospitals A, B, and C. Hospital characteristics are described in Table 1. Each hospital had in place an EHR with free text clinical documents containing details about the patients that were unlikely to appear in other coded, administrative datasets. At the time the study was conducted, Hospital A was using a homegrown EHR called CareWeb which had been in use since 1998. Hospital B was using the Cerner PowerChart system, and Hospital C had clinical documents from both an Epic EHR as well as their older McKesson system which Epic had replaced. This study was approved by each hospital’s local institutional review board. Waivers of informed consent and HIPAA waivers were granted by each institution’s review board to allow medical records review without written informed consent. Written informed consent was sought from those eligible to participate in the broader quality of life study.

**Table 1:** Hospital characteristics and results of two approaches for DSD case ascertainment at three children’s hospitals.

	Hospital A	Hospital B	Hospital C
**Hospital Characteristics**			
Hospital beds	300	254	245
Inpatient admissions	9,000	14,000	11,000
Surgical procedures	10,000	15,000	17,000
**Traditional Approach**			
Cases initially identified	NP	30	16
Final chart review			
Uncertain eligibility	NP	2	1
Not eligible	NP	0	1
Confirmed eligible	NP	28	14
**Informatics Approach** *			
ICD-9 cases	737	2,868	3,557
ICD-9 cases + text matches	728	2,742	2,550
Initial chart review	81	153	260
Final chart review			
Uncertain eligibility	6	13	18
Not eligible	16	63	16
Confirmed eligible	59	77	226

NP  =  not performed.

*At Hospital A, all cases were reviewed using the search engine EMERSE and it was not necessary to run a database query for keyword matching.

Probands in the study were determined to be eligible (or ruled ineligible) using a complex set of criteria based on information that is not routinely captured using coded or administrative data. Rather, the salient details are often captured only in the free text (i.e., dictated or typed) notes. For example, the study included patients with proximal hypospadias and either unilateral or bilateral undescended testes. To find such patients using administrative data, both hypospadias and undescended testes would have to be coded independently (which often does not occur), whereas the narrative descriptions more likely capture these details. Additionally, patients had to be between 0 and 81 months of age, without signs of significant developmental delay, and come from an English-speaking family. All institutions identified cases born during a seven-year time period. During each step of the patient identification process, study teams at each institution recorded the number of cases identified so that we could compare the capture rate of two distinct approaches for case ascertainment, described below.

### Case Ascertainment – Traditional Approach

The study was initiated at Hospital A. Collaborators at Hospitals B and C were provided with a detailed guide and instructions for identifying eligible patients along with a case-ascertainment log to record various patient characteristics for further review. Both institutions were asked to identify eligible patients for the study using their traditional approaches for cohort identification.

At Hospital B, a Master’s level genetic counselor, and an active member of the multidisciplinary DSD clinical team, gathered names of patients referred to their clinic. At Hospital C, a Master’s level research nurse coordinator, similarly a member of the local DSD clinical team, reviewed a database separate from the EHR containing a list of patients and other clinical data maintained by one subspecialty involved in the clinical management of patients with DSD. Cases in this database were identified using ICD-9 codes and then the charts were read for details about inclusion and exclusion criteria. Hospital A did not use the traditional approach because the research team recognized the limitations of these strategies and because the study team had access to specialized software for searching the clinical documents, discussed in the following section.

### Case Ascertainment – Informatics Approach

The goal of the informatics approach was to replicate the search process supported by the search engine software (EMERSE) at Hospital A [32]–[35]. EMERSE, the Electronic Medical Record Search Engine, accepts a set of patient medical record numbers and a set of search terms as input. It then scans each patient’s documents, and returns the results in a format ideal for chart review. The display includes highlighting all relevant terms in the documents. A typical workflow for a study utilizing EMERSE is to first identify a potential patient cohort using structured data sources including ICD-9 codes, procedure codes, clinical schedules, or existing registries. Cohort identification tools for structured data, such as the i2b2 Workbench, are already in widespread use [36]. The use of ICD-9 codes can often generate lists much larger than those obtained through traditional approaches by casting a wider net to capture patients that might otherwise be missed. The main downside is that the codes are often very non-specific and inaccurately assigned. However, these larger lists can easily be reviewed with tools such as EMERSE.

Using the patient list identified through structured data sources, a user can then enter any number of keywords or phrases into the system, and EMERSE will then search for those phrases in the free text clinical documentation. With EMERSE, the complexity of the searching is hidden from end users, who do not need to know how to use advanced computational tools to complete their work.

**Figure 1:**
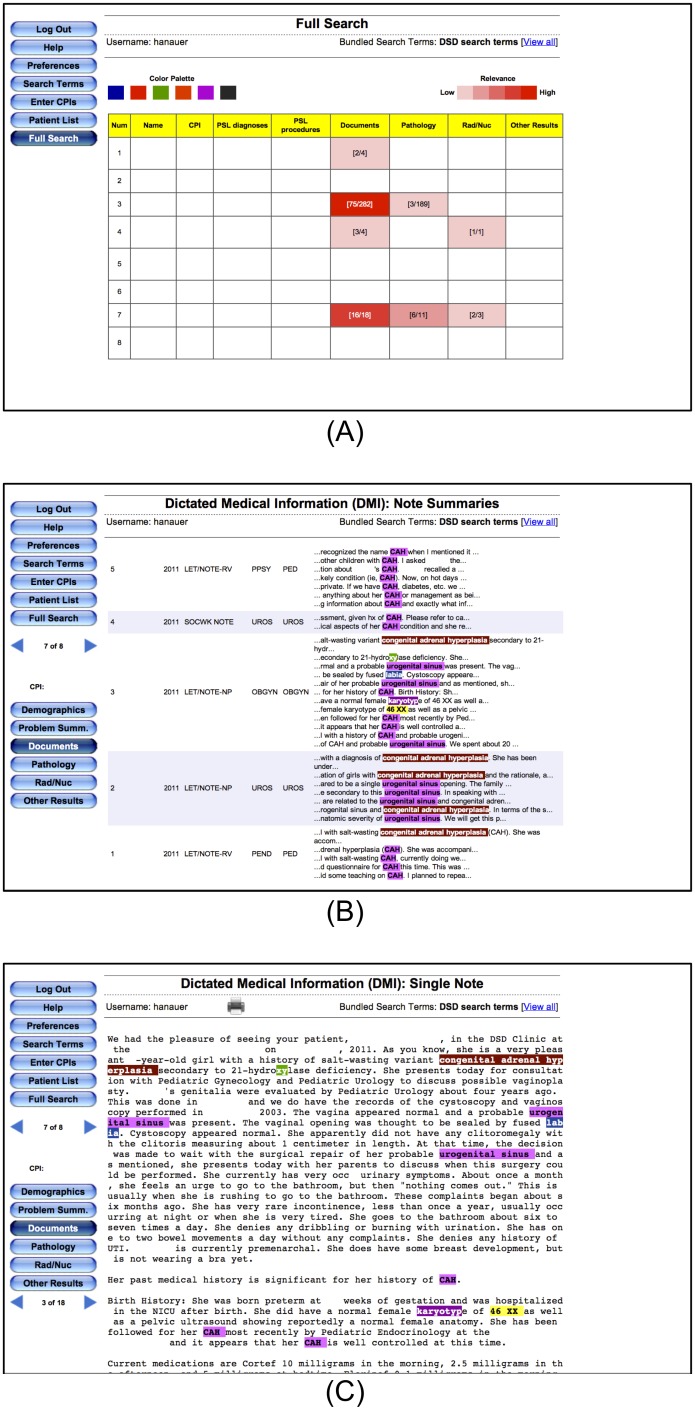
Search results for the DSD terms using EMERSE at Hospital A. All identifiers have been redacted. Search results are presented in three views for assisting with rapid review. (A) The highest level displays a ‘heat map’ of results, with rows representing different patients and columns representing different components of the medical record. Sections with “hits” are highlighted; darker colors represent more documents with a hit. (B) Clicking on a cell in the heat map displays a summary of the documents for a specific patient, with summaries around the hits shown. Here alternating shaded rows represents a single document for a patient. (C) Clicking on a specific document summary brings up the full document with all search terms still highlighted. Note that the “xy” highlighted in ‘hydroxylase’ is actually a false positive result, as it was intended to identify karyotypes. Options were available in EMERSE to reduce this type of false hit, but they were not used in this study.

EMERSE has been used for a wide variety of studies that have been published in peer-reviewed journals including clinical [33], [37]–[41] as well as translational research [42], [43]. The use of EMERSE has allowed investigators at our institution to carry out many studies (both for case ascertainment and data abstraction) that were not practical prior to its implementation. One study that leveraged EMERSE to automate the identification of the postoperative surgical complications myocardial infarction and pulmonary embolus was able to achieve a sensitivity of 100% and 93%, respectively with specificities of 93% and 96%, respectively [44]. Another study that used EMERSE for eligibility determination in a depression study found significant time savings while maintaining clinical accuracy [33]. Figure 1 displays three screen shots from the EMERSE search engine as it was used for the current DSD case ascertainment study.

In addition to using their traditional approaches for case ascertainment, the two institutions that did not use EMERSE (Hospitals B and C), were asked to identify an initial cohort of potential patients using ten ICD-9 codes (Table 2). This list was intentionally broad, meant to initially favor sensitivity over specificity. Because both institutions also had clinical notes in electronic, free text format, we also provided a list of 26 search terms (Table 3) that could be used to narrow the list of eligible patients initially identified using the ICD-9 codes. That is, if any of the terms appeared in a patient’s note, that patient was a potential candidate. Furthermore, these terms, when highlighted within the documents, made chart review highly efficient. The search terms themselves were not meant to find mention of specific diagnoses (e.g., Swyer syndrome, complete androgen insensitivity syndrome, etc) but rather high-level physical and genetic testing characteristics that can help identify features consistent with a DSD. This is because many times the actual diagnosis may not be clearly described in the clinical notes but the phenotypic characteristics will provide supporting evidence for such a diagnosis. The diagnoses falling under the umbrella of DSD are complex [45], so using a more general approach without excessively specific search terms was determined by the DSD team to be of greatest value.

**Table 2:** ICD-9 codes used to identify an initial cohort of potentially eligible patients.

ICD-9 code	Description
752	Congenital anomalies of genital organs
752.4	Abnormalities of cervix, vagina, and external female genitalia
752.40	Unspecified anomaly of cervix, vagina, and external female genitalia
752.49	Other anomalies of cervix, vagina, and external female genitalia
752.61	Hypospadias
752.64	Micropenis
752.69	Other penile anomalies
752.7	Indeterminate sex and pseudohermaphroditism
255.2*	Adrenogenital disorders (eg, congenital adrenal hyperplasia)
259.5	Androgen Insensitivity Syndrome (AIS) (partial and complete)

*This specific code was restricted to female patients only.

**Table 3:** Keywords used to highlight relevant information in the clinical documents identified from the ICD-9 search.

DSD Keywords	
46 XX	CAH
46 XY	congenital adrenal hyperplasia
46-XX	gonad
46-XY	hypospad
XO	labia
XX	mosaic
XY	penile
ambig	penis
chordee	phall
karyotyp	prader
penoscrotal	urogenital sinus
perineal	viriliz
severe hypospad	
undescended	

Keywords and phrases in the left column were used in conjunction with the ICD-9 code (752.61) for hypospadias; whereas those in both columns were used in conjunction with the balance (ie, non-hypospadias) of ICD-9 codes listed in Table 2. Note that because of the searching process, a keyword search for a term such as “gonad” would also identify terms such as “gonads”, “gonadic”, and “gonadal”.

**Figure 2:**
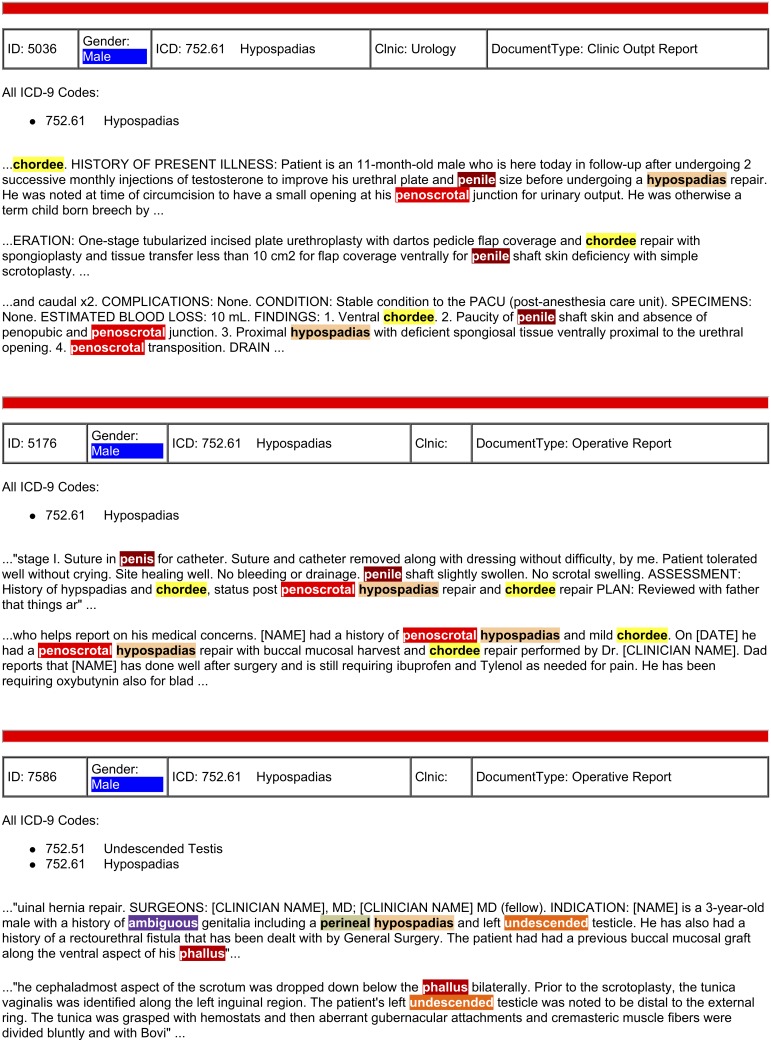
Search results for the DSD terms at Hospital B. These snippets of text with DSD keywords highlighted are from three patients that were identified using the generic ICD-9 code 752.61 (hypospadias). By reviewing the highlighted text, the study team was able to determine that these patients were likely to exhibit a DSD rather than isolated hypospadias.

Both hospitals (B and C) obtained assistance from their local medical information technology (IT) teams to run the database queries and obtain the necessary data for chart review. They first used the set of ICD-9 codes, date ranges, and dates of birth to search their administrative databases to identify a large, initial cohort of potentially eligible patients. The IT teams were then instructed to further identify potential cases from this larger cohort who had at least one of the keyword terms in their notes (Table 3). They were instructed to search in a manner that matched any subset of text. Thus, a search for “karyotyp” would identify words such as “karyotype,” ”karyotyped,” “karyotypes,” and “karyotyping.” Sophisticated search engines can sometimes handle these variations in word endings (referred to as stemming), but traditional database searches do not. Such database searches typically use structured query language (SQL) queries using the LIKE command for string matching (e.g., SELECT patient WHERE text LIKE ‘%karyotyp%’). Regular expressions, a powerful approach for complex pattern matching in text, can be used for more advanced searching in databases, but requires an additional level of expertise. To ease the review of cases, we asked that the keywords be highlighted in the documents so that the chart review could be more efficient, as was the case for EMERSE at Hospital A; this required additional computer code to be written. These keywords were displayed as snippets, or excerpts, with approximately 3 to 5 additional words on either side to provide context yet maintain brevity for rapid reviewing. An example of how these snippets from Hospital A were displayed in EMERSE can be seen in Figure 1B. Similarly, text snippets that were reviewed from data obtained from Hospital B are shown in Figure 2 for comparison. While not completely identical, the SQL used in hospitals B and C was similar to the SQL that was being used at hospital A by the EMERSE system in production use at that time. It is worth pointing out that while the ease of use of EMERSE would help users save time, our goal in this study was not to compare time efficiency but rather to compare the completeness of patient case identification using the standard approaches versus the more advanced approach that leveraged SQL.

### Manual Chart Review

All cases identified via the traditional and informatics approaches were manually reviewed by trained research assistants, MG, and DES for additional inclusion and exclusion criteria. Because of the larger number of cases initially identified using the informatics approach, the chart review was performed in two stages. In the initial chart review, ineligible cases (based on requirements of the parent study) were removed such as those with developmental delay, isolated distal hypospadias, cloacal exstrophy, Turner syndrome, and Klinefelter syndrome. The final chart review required a more detailed inspection of cases including a review of a description of the genitals, laboratory values, and other diagnoses.

## Results

The numbers of cases identified through both the traditional and informatics approaches are listed in Table 1. While the traditional approach was not used at Hospital A, this approach yielded only a relatively small number of cases at the other two hospitals, with 28 and 14 cases identified at Hospital B and C, respectively. By contrast, the number of cases ascertained using the informatics approach was substantially higher: 77 cases were identified at Hospital B using the informatics approach, representing a nearly three-fold increase in study patients. For Hospital C the informatics approach identified 226 patients eligible for the study, a 16-fold increase in study patients.

Using ICD-9 codes as an initial screen to identify patients yielded many more potential cases that had to be ruled out. At Hospital A, 8.0% of the ICD-9 cases were eventually found to be truly eligible, compared to 2.7% for Hospital B and 6.4% for Hospital C.

**Figure 3:**
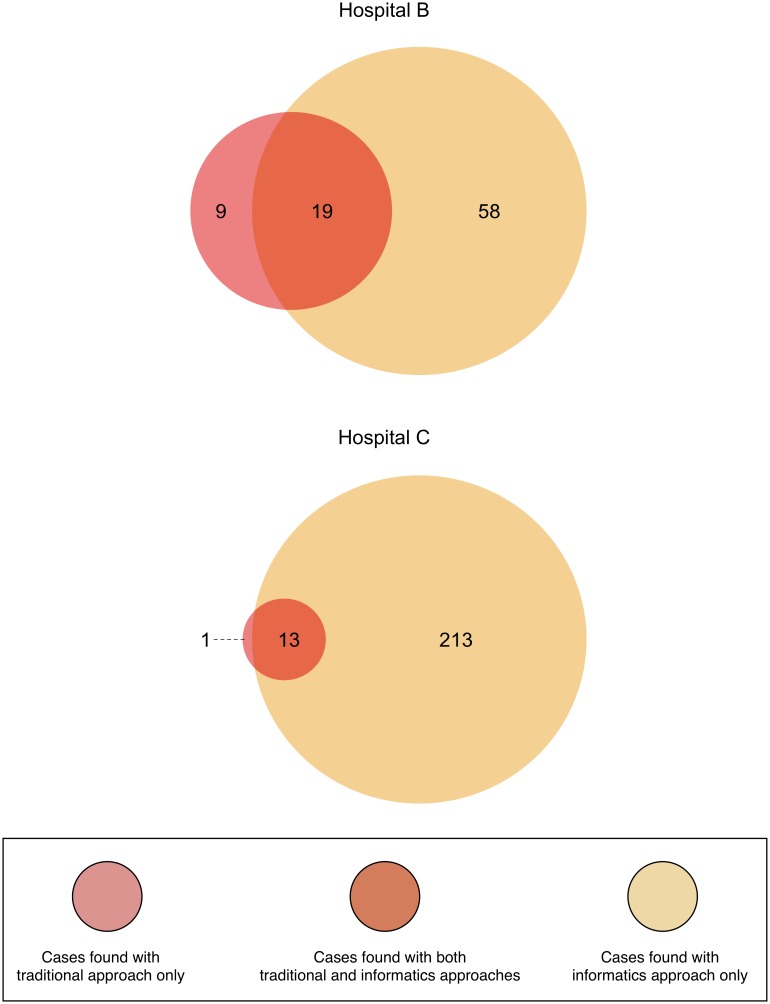
Cases of DSD found using the traditional and informatics approaches. Cases of DSD found using the traditional and informatics approaches, showing the overlap of cases found jointly by both approaches and those found distinctly with either approach. Additional details about the cases can be found in the Supporting Information tables.

Venn Diagrams displaying the number of cases found jointly by the traditional and informatics approaches, and those cases only ascertained by one of the two approaches are shown in Figure 3. The informatics approach identified many more cases than did the traditional approach, and many of those patients would not have been identified using only the traditional approach. Conversely, the traditional approach identified a small number of cases that the informatics approach missed. At Hospital B, nine cases were identified only with the traditional approach whereas only one such case was identified with the traditional approach at Hospital C.

Additional details about the DSD cases that were verified as having a true DSD at both Hospitals B and C are provided as Supporting Information tables. Tables S1, S2, S3, S4, S5, and S6 report the combined counts for both Hospitals B and C. Tables S7, S8, S9, S10, S11 and S12 report the counts for Hospital B, and Tables S13, S14, S15, S16, S17 and S18 reports the counts for Hospital C. For each group of tables, the counts are broken down by various regions of the Venn diagrams as described in the manuscript.

One case of complete androgen insensitivity syndrome (CAIS) was identified using the traditional approach without the use of an ICD-9 code, so this was not detected using the informatics approach. For all of the other codes, the informatics approach found the same number or more cases (Table S19), ranging from 1 case each of ICD-9 code 752.4 (Unspecified Congenital Anomaly of Cervix, Vagina, and External Female Genitalia) via both the traditional and informatics approaches to a 25-fold increase in the number of cases of the non-specific ICD-9 code 752.69 (Other Penile Anomalies) found with the informatics approach.

## Discussion

Rare diseases can often be heterogeneous in their presentation [46]–[48], and it can be challenging to identify affected persons in a comprehensive and systematic manner [49]. Thus, the difficulty in obtaining adequate patient cohorts may lead to less rigorous recruitment practices which, itself, can introduce bias [50]. Case ascertainment in DSD has historically been challenging due to a lack of standardized definitions of what actually constitutes a DSD. Disagreements about definitions persist [51], [52], suggesting that additional focus should be placed on lessening the impact of other shortcomings in the cohort identification process that could lead to non-representative samples. As registries are being developed to comprehensively capture cases of rare diseases, including DSD [53], it will become increasingly important to ensure that the patients are included using unbiased strategies [54].

The traditional approaches for case identification in the multi-institutional collaboration described in the current study were varied, but seemed to mimic traditional manual searches through paper charts, which are neither efficient nor comprehensive. But when we standardized the approaches by using techniques that are enabled through the use of EHRs, the number of patients identified was substantially higher. This suggests that while many diseases are rare, they might not always be as rare as it may seem when a more comprehensive approach is used to identify patients. Further, this more inclusive approach is likely to increase statistical power from larger study samples and reduce selection bias that may occur when single clinics or clinicians “hand-pick” study participants. A strength of our study was that we demonstrated the effectiveness of the informatics approach using clinical notes generated through multiple EHRs. Thus, even though there may be variations in hospitals, or even among clinical groups with respect to documentation practices and conventions, the informatics approach was still able to identify more cases than the traditional approach.

ICD-9 codes are often used, but insufficient, for identifying rare diseases [19], in large part because there is not a specific code for each disease or phenotype. This was also evident in our study where we initially used ICD-9 codes but then reduced the many candidate cases through additional keyword searches. The transition in the U.S. from ICD-9 to ICD-10 codes has the potential to improve the way diagnoses are coded, but it remains to be seen how precise clinicians will be when using the codes. For example, the ICD-10 code Q54.2 (“hypospadias, penoscrotal”) would potentially be indicative of a patient with a DSD, but if clinicians choose to use the more generic and much less specific code Q54.9 (“hypospadias, unspecified”), then further review of the records will still be required to confirm that the case meets definitional requirements of the category DSD. In our current study, we found patients with hypospadias to be very challenging in terms of identifying DSD cases since it has such a broad phenotype. A recent study conducted in Norway (where ICD-10 has been in use since 1997) [55] used a combination of ICD codes to identify patients but still required a manual chart review to verify and systematically identify all patients with congenital adrenal hyperplasia [56]. This should not be surprising given the known challenges of accurately assigning codes for both the ICD-9 [16]–[18] and ICD-10 systems [57]–[63]. One study specifically acknowledged that “the implementation of ICD-10 coding has not significantly improved the quality of administrative data relative to ICD-9-CM.” [64].

The keywords used in our study did not by themselves narrow down the cases to a significant extent, but by highlighting them in the text it allowed our reviewers to focus on those specific sections of text and allowed them to rapidly identify the key concepts to help make the eligibility determination. That is why the number of cases identified with ICD-9 alone did not differ much from those identified with ICD-9 plus the text matches. However, the review of the cases that occurred because of the text matches allowed for the cases to be effectively narrowed to only appropriate ones. Additionally, because we were able to identify and highlight keywords across all documents for each patient, we were able to ensure a more comprehensive approach to chart review while maintaining efficiency. Future work should explore the use of negation or exclusions (e.g., not highlighting ‘labia’ in the context of ‘labia were normal in appearance’) as a way to potentially reduce the number of false positive terms highlighted. EMERSE already supports a simple version of exclusions, but it was not used in this study. Additionally, comparison of this informatics-based, but relatively simple, approach to more advanced computational natural language processing techniques would be worthwhile.

While our informatics approach increased the number of cases identified, there were a small number of cases that were missed and were only identified using the traditional approaches. One patient, for example, was seen only at an affiliated satellite clinic that had its own EHR, and had never been seen at the main hospital from which the study recruitment was being conducted. The clinical DSD team was nonetheless aware of the patient and identified them for inclusion. This raises an issue that even when using comprehensive approaches for identifying patients at large health centers, additional efforts may be required to comprehensively identify the overall population of patients with rare diseases in a given region.

While the three study settings were not precisely comparable in size and volume of procedures, all were full tertiary care children’s hospitals with comprehensive specialty services including both medical and surgical services. All three have specialty services that diagnose and treat patients with DSD, and conduct research on DSD. It should be noted that at Hospital A, we used a well-established tool, EMERSE, that had functionality we attempted to replicate at the other two institutions, and with modest effort we were able to implement some of the core features of EMERSE at both institutions, yielding a much larger number of cases identified. EMERSE provides a simple user interface that makes running searches quick and efficient, but our goal in this paper was to focus on the portability and efficacy of this approach at other institutions rather than the usability or speed of EMERSE itself. The method proved to be easily reproducible at both other institutions with help from the IT teams. Further, if EMERSE itself had been installed at the two other institutions, the analyses could have been carried out without any help from the IT groups once the software installation was complete.

The version of EMERSE used at Hospital A for the study described here has been replaced by a newer version that integrates data from an older, locally developed EHR system as well as clinical documents from the new vendor system (Epic). EMERSE is available at no cost for academic use at other institutions, and interested investigators can contact the authors for additional information about the system. A demonstration version of the system is available at http://project-emerse.org.

Our approach for case ascertainment should be applicable to other rare disease research, and may help standardize the way in which cases are identified across multiple institutions. To achieve this goal, we suggest that future studies provide additional details describing their case ascertainment approaches to reduce the likelihood that selection biases are responsible for variability in results across studied populations. Detailed reporting of methodology is already required for many clinical trials using frameworks such as the Consolidated Standards of Reporting Trials (CONSORT) [65]. One way this type of reporting could be improved would be for investigators to provide the list of keywords or search terms used (Table 3). Indeed, other studies that have used similar approaches have listed the search terms used [31], [33], [38], [40], [41] and some authors have noted the benefits of using systems such as EMERSE to ensure standardized and reproducible results by searching in a systematic and unbiased manner [66], [67].

## Conclusion

To the best of our knowledge, this is the first time that an informatics-based approach has been used for the systematic identification of cases of DSD in an electronic health record. The results of our study demonstrate that traditional approaches for case ascertainment in DSD are limited and may introduce bias. The use of EHRs alone may not lead to a reduction in this bias unless additional strategies are utilized with these systems to more comprehensively identify patients for study inclusion. Applying more rigorous and reproducible informatics-based approaches for case ascertainment is now feasible and should be considered as research teams recruit patients for studies of rare diseases.

## Supporting Information

Table S1 **Hospital B + Hospital C: Patients identified by all methods.**
(PDF)

Table S2 **Hospital B + Hospital C: Patients identified by Standard Method.**
(PDF)

Table S3 **Hospital B + Hospital C: Patients identified by Informatics.**
(PDF)

Table S4 **Hospital B + Hospital C: Patients identified only by Standard Method.**
(PDF)

Table S5 **Hospital B + Hospital C: Patients identified only by Informatics.**
(PDF)

Table S6 **Hospital B + Hospital C: Patients identified by Informatics and Standard Method.**
(PDF)

Table S7 **Hospital B: Patients identified by all methods.**
(PDF)

Table S8 **Hospital B: Patients identified by Standard Method.**
(PDF)

Table S9 **Hospital B: Patients identified by Informatics.**
(PDF)

Table S10 **Hospital B: Patients identified only by Standard Method.**
(PDF)

Table S11 **Hospital B: Patients identified only by Informatics.**
(PDF)

Table S12 **Hospital B: Patients identified by Informatics and Standard Method.**
(PDF)

Table S13 **Hospital C: Patients identified by all methods.**
(PDF)

Table S14 **Hospital C: Patients identified by Standard Method.**
(PDF)

Table S15 **Hospital C: Patients identified by Informatics.**
(PDF)

Table S16 **Hospital C: Patients identified only by Standard Method.**
(PDF)

Table S17 **Hospital C: Patients identified only by Informatics.**
(PDF)

Table S18 **Hospital C: Patients identified by Informatics and Standard Method.**
(PDF)

Table S19 **Collapsed/merged ICD-9 codes for Hospitals B and C combined.**
(PDF)
