# Supplementary material for: Unbiased Identification of Patients with Disorders of Sex Development
Source: PLoS One. 2014 Sep 30;9(9):e108702. doi: 10.1371/journal.pone.0108702 (PMC4182545; doi:10.1371/journal.pone.0108702)
Supplement: Table S1 — Hospital B + Hospital C: Patients identified by all methods. (PDF) [file pone.0108702.s001.pdf]

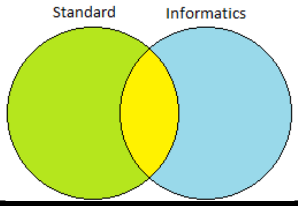

Table S1. Hospital B + Hospital C: Patients identified by all methods	n
255.2 ADRENOGENITAL DISORDERS	55
255.2 CAH (CONGENITAL ADRENAL HYPERPLASIA)	34
255.2 CONGENITAL ADRENAL HYPERPLASIA, TYPE 1	6
255.2 ADRENAL HYPERPLASIA, CONGENITAL	3
255.2 ADRENAL HYPERPLASIA SYNDROME, CONGENITAL	1
255.2 11 BETA-HYDROXYLASE DEFICIENCY	1
255.2 CONGENITAL ADRENAL CORTICAL HYPERPLASIA	1
259.5 ANDROGEN INSENSITVTY SYN	1
259.5 PARTIAL ANDROGEN INSENSITIVITY	1
259.5 ANDROGEN INSENSITIVITY SYNDROME	2
752.4 Unspecified Congenital Anomaly of Cervix, Vagina, and External Female Genitalia	2
752.49 CERVIX/FEM GEN ANOM NEC	7
752.49 CERVIX/FEM GEN ANOM	1
752.49 ATRESIA OF VAGINA	1
752.49 VAGINA, ABSENCE OF	1
752.51 Undescended Testis	23
752.61 HYPOSPADIAS	197
752.61 HYPOSPADIA	5
752.61 Hypospadias, male	4
752.64 MICROPENIS	54
752.64 MICROPHALLUS	6
752.69 PENILE ANOMALIES NEC	19
752.69 OTHER PENILE ANOMALIES	31
752.69 PENILE ANOMALY	2
752.69 ANOMALY OF PENIS	1
752.7 INTERSEXUALITY	1
752.7 INDETERMINATE SEX	21
752.7 INDETERMINATE SEX AND PSEUDOHERMAPHRODITISM	62
752.7 HERMAPHRODITISM	1
752.7 PERSISTENT MULLERIAN DUCT SYNDROME	1
752.7 DISORDER OF SEXUAL DIFFERENTIATION	1
752.7 Ambiguous genitalia	9
752.7 GONADAL DYSGENESIS, 46,XY	4
CAIS complete androgen insensitivity listed without an ICD9 code	1
