# Supplementary material for: Unbiased Identification of Patients with Disorders of Sex Development
Source: PLoS One. 2014 Sep 30;9(9):e108702. doi: 10.1371/journal.pone.0108702 (PMC4182545; doi:10.1371/journal.pone.0108702)
Supplement: Table S5 — Hospital B + Hospital C: Patients identified only by Informatics. (PDF) [file pone.0108702.s005.pdf]

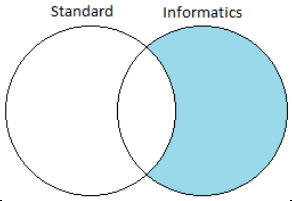

Table S5. Hospital B + Hospital C: Patients identified only by Informatics	n
255.2 ADRENOGENITAL DISORDERS	40
255.2 CAH (CONGENITAL ADRENAL HYPERPLASIA)	25
255.2 CONGENITAL ADRENAL HYPERPLASIA, TYPE 1	4
255.2 ADRENAL HYPERPLASIA, CONGENITAL	2
255.2 ADRENAL HYPERPLASIA SYNDROME, CONGENITAL	0
255.2 11 BETA-HYDROXYLASE DEFICIENCY	1
255.2 CONGENITAL ADRENAL CORTICAL HYPERPLASIA	1
259.5 ANDROGEN INSENSITVTY SYN	1
259.5 PARTIAL ANDROGEN INSENSITIVITY	1
259.5 ANDROGEN INSENSITIVITY SYNDROME	1
752.4 Unspecified Congenital Anomaly of Cervix, Vagina, and External Female Genitalia	1
752.49 CERVIX/FEM GEN ANOM NEC	4
752.49 CERVIX/FEM GEN ANOM	1
752.49 ATRESIA OF VAGINA	1
752.49 VAGINA, ABSENCE OF	1
752.51 Undescended Testis	13
752.61 HYPOSPADIAS	173
752.61 HYPOSPADIA	5
752.61 Hypospadias, male	1
752.64 MICROPENIS	50
752.64 MICROPHALLUS	5
752.69 PENILE ANOMALIES NEC	19
752.69 OTHER PENILE ANOMALIES	29
752.69 PENILE ANOMALY	2
752.69 ANOMALY OF PENIS	1
752.7 INTERSEXUALITY	1
752.7 INDETERMINATE SEX	14
752.7 INDETERMINATE SEX AND PSEUDOHERMAPHRODITISM	41
752.7 HERMAPHRODITISM	0
752.7 PERSISTENT MULLERIAN DUCT SYNDROME	1
752.7 DISORDER OF SEXUAL DIFFERENTIATION	1
752.7 Ambiguous genitalia	1
752.7 GONADAL DYSGENESIS, 46,XY	1
CAIS complete androgen insensitivity listed without an ICD9 code	0
