# Supplementary material for: Unbiased Identification of Patients with Disorders of Sex Development
Source: PLoS One. 2014 Sep 30;9(9):e108702. doi: 10.1371/journal.pone.0108702 (PMC4182545; doi:10.1371/journal.pone.0108702)
Supplement: Table S8 — Hospital B: Patients identified by Standard Method. (PDF) [file pone.0108702.s008.pdf]

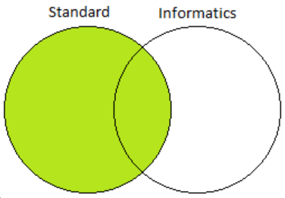


| Table S8. Hospital B: Patients identified by Standard Method | n |
|---|---|
| 255.2 ADRENOGENITAL DISORDERS | 7 |
| 255.2 CAH (CONGENITAL ADRENAL HYPERPLASIA) | 3 |
| 255.2 CONGENITAL ADRENAL HYPERPLASIA, TYPE 1 | 0 |
| 255.2 ADRENAL HYPERPLASIA, CONGENITAL | 0 |
| 255.2 ADRENAL HYPERPLASIA SYNDROME, CONGENITAL | 0 |
| 255.2 11 BETA-HYDROXYLASE DEFICIENCY | 0 |
| 255.2 CONGENITAL ADRENAL CORTICAL HYPERPLASIA | 0 |
| 259.5 ANDROGEN INSENSITVTY SYN | 0 |
| 259.5 PARTIAL ANDROGEN INSENSITIVITY | 0 |
| 259.5 ANDROGEN INSENSITIVITY SYNDROME | 1 |
| 752.4 Unspecified Congenital Anomaly of Cervix, Vagina, and External Female Genitalia | 1 |
| 752.49 CERVIX/FEM GEN ANOM NEC | 0 |
| 752.49 CERVIX/FEM GEN ANOM | 0 |
| 752.49 ATRESIA OF VAGINA | 0 |
| 752.49 VAGINA, ABSENCE OF | 0 |
| 752.51 Undescended Testis | 10 |
| 752.61 HYPOSPADIAS | 18 |
| 752.61 HYPOSPADIA | 0 |
| 752.61 Hypospadias, male | 3 |
| 752.64 MICROPENIS | 3 |
| 752.64 MICROPHALLUS | 0 |
| 752.69 PENILE ANOMALIES NEC | 0 |
| 752.69 OTHER PENILE ANOMALIES | 2 |
| 752.69 PENILE ANOMALY | 0 |
| 752.69 ANOMALY OF PENIS | 0 |
| 752.7 INTERSEXUALITY | 0 |
| 752.7 INDETERMINATE SEX | 0 |
| 752.7 INDETERMINATE SEX AND PSEUDOHERMAPHRODITISM | 12 |
| 752.7 HERMAPHRODITISM | 0 |
| 752.7 PERSISTENT MULLERIAN DUCT SYNDROME | 0 |
| 752.7 DISORDER OF SEXUAL DIFFERENTIATION | 0 |
| 752.7 Ambiguous genitalia | 8 |
| 752.7 GONADAL DYSGENESIS, 46,XY | 3 |
| CAIS complete androgen insensitivity listed without an ICD9 code | 1 |
