# Supplementary material for: Unbiased Identification of Patients with Disorders of Sex Development
Source: PLoS One. 2014 Sep 30;9(9):e108702. doi: 10.1371/journal.pone.0108702 (PMC4182545; doi:10.1371/journal.pone.0108702)
Supplement: Table S11 — Hospital B: Patients identified only by Informatics. (PDF) [file pone.0108702.s011.pdf]

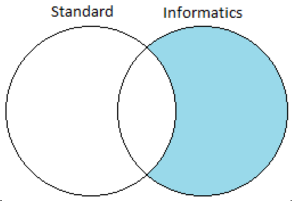

Table S11. Hospital B: Patients identified only by Informatics	n
255.2 ADRENOGENITAL DISORDERS	7
255.2 CAH (CONGENITAL ADRENAL HYPERPLASIA)	3
255.2 CONGENITAL ADRENAL HYPERPLASIA, TYPE 1	0
255.2 ADRENAL HYPERPLASIA, CONGENITAL	0
255.2 ADRENAL HYPERPLASIA SYNDROME, CONGENITAL	0
255.2 11 BETA-HYDROXYLASE DEFICIENCY	0
255.2 CONGENITAL ADRENAL CORTICAL HYPERPLASIA	0
259.5 ANDROGEN INSENSITVTY SYN	0
259.5 PARTIAL ANDROGEN INSENSITIVITY	0
259.5 ANDROGEN INSENSITIVITY SYNDROME	0
752.4 Unspecified Congenital Anomaly of Cervix, Vagina, and External Female Genitalia	1
752.49 CERVIX/FEM GEN ANOM NEC	0
752.49 CERVIX/FEM GEN ANOM	0
752.49 ATRESIA OF VAGINA	0
752.49 VAGINA, ABSENCE OF	0
752.51 Undescended Testis	13
752.61 HYPOSPADIAS	36
752.61 HYPOSPADIA	0
752.61 Hypospadias, male	1
752.64 MICROPENIS	8
752.64 MICROPHALLUS	0
752.69 PENILE ANOMALIES NEC	0
752.69 OTHER PENILE ANOMALIES	5
752.69 PENILE ANOMALY	0
752.69 ANOMALY OF PENIS	0
752.7 INTERSEXUALITY	0
752.7 INDETERMINATE SEX	0
752.7 INDETERMINATE SEX AND PSEUDOHERMAPHRODITISM	11
752.7 HERMAPHRODITISM	0
752.7 PERSISTENT MULLERIAN DUCT SYNDROME	0
752.7 DISORDER OF SEXUAL DIFFERENTIATION	0
752.7 Ambiguous genitalia	1
752.7 GONADAL DYSGENESIS, 46,XY	0
CAIS complete androgen insensitivity listed without an ICD9 code	0
