# Supplementary material for: Unbiased Identification of Patients with Disorders of Sex Development
Source: PLoS One. 2014 Sep 30;9(9):e108702. doi: 10.1371/journal.pone.0108702 (PMC4182545; doi:10.1371/journal.pone.0108702)
Supplement: Table S14 — Hospital C: Patients identified by Standard Method. (PDF) [file pone.0108702.s014.pdf]

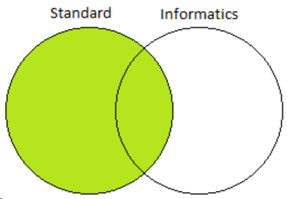

Table S14. Hospital C: Patients identified by Standard Method	n
255.2 ADRENOGENITAL DISORDERS	8
255.2 CAH (CONGENITAL ADRENAL HYPERPLASIA)	6
255.2 CONGENITAL ADRENAL HYPERPLASIA, TYPE 1	2
255.2 ADRENAL HYPERPLASIA, CONGENITAL	1
255.2 ADRENAL HYPERPLASIA SYNDROME, CONGENITAL	1
255.2 11 BETA-HYDROXYLASE DEFICIENCY	0
255.2 CONGENITAL ADRENAL CORTICAL HYPERPLASIA	0
259.5 ANDROGEN INSENSITVTY SYN	0
259.5 PARTIAL ANDROGEN INSENSITIVITY	0
259.5 ANDROGEN INSENSITIVITY SYNDROME	0
752.4 Unspecified Congenital Anomaly of Cervix, Vagina, and External Female Genitalia	0
752.49 CERVIX/FEM GEN ANOM NEC	3
752.49 CERVIX/FEM GEN ANOM	0
752.49 ATRESIA OF VAGINA	0
752.49 VAGINA, ABSENCE OF	0
752.51 Undescended Testis	0
752.61 HYPOSPADIAS	6
752.61 HYPOSPADIA	0
752.61 Hypospadias, male	0
752.64 MICROPENIS	1
752.64 MICROPHALLUS	1
752.69 PENILE ANOMALIES NEC	0
752.69 OTHER PENILE ANOMALIES	0
752.69 PENILE ANOMALY	0
752.69 ANOMALY OF PENIS	0
752.7 INTERSEXUALITY	0
752.7 INDETERMINATE SEX	7
752.7 INDETERMINATE SEX AND PSEUDOHERMAPHRODITISM	9
752.7 HERMAPHRODITISM	1
752.7 PERSISTENT MULLERIAN DUCT SYNDROME	0
752.7 DISORDER OF SEXUAL DIFFERENTIATION	0
752.7 Ambiguous genitalia	0
752.7 GONADAL DYSGENESIS, 46,XY	0
CAIS complete androgen insensitivity listed without an ICD9 code	0
