# Supplementary material for: Unbiased Identification of Patients with Disorders of Sex Development
Source: PLoS One. 2014 Sep 30;9(9):e108702. doi: 10.1371/journal.pone.0108702 (PMC4182545; doi:10.1371/journal.pone.0108702)
Supplement: Table S19 — Collapsed/merged ICD-9 codes for Hospitals B and C combined. (PDF) [file pone.0108702.s019.pdf]

Table S19. Collapsed/merged ICD-9 codes for Hospitals B and C combined

ICD-9 code	Found by traditional approach (data from Table S2)	Found by informatics approach only (data from Table S5)	Ratio of cases informatics only / traditional approach
255.2	28	73	2.6
259.5	1	3	3.0
752.4	1	1	1.0
752.49	3	7	2.3
752.51	10	13	1.3
752.61	27	179	6.6
752.64	5	55	11.0
752.69	2	51	25.5
752.7	40	60	1.5
